# Response of Patch Characteristics of *Carex alatauensis* S. R. Zhang to Establishment Age in Artificial Grasslands on the Qinghai–Tibet Plateau, China

**DOI:** 10.3390/plants14152257

**Published:** 2025-07-22

**Authors:** Liangyu Lyu, Chao Wang, Pei Gao, Fayi Li, Qingqing Liu, Jianjun Shi

**Affiliations:** 1 Academy of Animal Husbandry and Veterinary Sciences, Qinghai University, Xining 810016, China; yb230909000074@qhu.edu.cn (L.L.); 912882398@qq.com (C.W.); y200954000466@qhu.edu.cn (P.G.); lfy99218@qhu.edu.cn (F.L.); yb220909000082@qhu.edu.cn (Q.L.); 2 Key Laboratory of Adaptive Management of Alpine Grassland, Xining 810016, China; 3 State Key Laboratory of Ecology and Plateau Agriculture and Animal Husbandry in Sanjiangyuan Jointly Established by the Ministry of Provincial Affairs, Qinghai University, Xining 810016, China

**Keywords:** alpine vegetation restoration, Cyperaceae, succession, plant community, soil nutrients

## Abstract

To clarify the ecological mechanisms underlying the succession of artificial grasslands to native alpine meadows and systematically reveal the patterns of ecological restoration in artificial grasslands in the Qinghai–Tibet Plateau, this study provides a theoretical basis for alpine meadow ecological restoration. In this study, artificial grassland and degraded grassland (CK) with different restoration years (20 years, 16 years, 14 years, and 2 years) in the Qinghai–Tibet Plateau were taken as research objects. We focused on the tillering characteristics, patch number, community structure evolution, and soil properties of the dominant species, *C. alatauensis,* and systematically explored the ecological restoration law by comparing and analyzing ecological indicators in different restoration years. The results showed the following: (1) With the extension of restoration years, the asexual reproduction ability of *C. alatauensis* was enhanced, the patches became large, and aboveground/underground biomass significantly accumulated. (2) Community structure optimization meant that the coverage and biomass of Cyperaceae plants increased with restoration age, while those of Poaceae plants decreased. The diversity of four species in 20A of restored grasslands showed significant increases (10.71–19.18%) compared to 2A of restored grasslands. (3) Soil improvement effect: The contents of soil organic carbon (SOC), total phosphorus (TP), nitrate nitrogen (NN), and available phosphorus (AP) increased significantly with the restoration years (in 20A, the SOC content in the 0–10 cm soil layer increased by 57.5% compared with CK), and the soil pH gradually approached neutrality. (4) In artificial grasslands with different restoration ages (20A, 16A, and 14A), significant or highly significant correlations existed between *C. alatauensis* tiller characteristics and community and soil properties. In conclusion, *C. alatauensis* in artificial grasslands drives population enhancement, community succession, and soil improvement through patch expansion.

## 1. Introduction

The Tibetan Plateau, the core ecological security barrier of China, contains 38 percent of the country’s total natural grassland area. Of this, alpine meadow is the most widely distributed grassland type, with the richest species diversity. It plays an irreplaceable ecological role in the regulation of the regional water cycle, the enhancement of carbon sink function, the stabilization of the climatic system, and the maintenance of biodiversity [1,2]. As a representative species of the Cyperaceae, *Carex alatauensis* S. R. Zhang has unique ecological and economic values: the plant is rich in protein, nitrogen-free leachate, and fat, with much higher nutritional value and palatability than Poaceae and Fabaceae forages. It can be efficiently utilized by yaks, Tibetan sheep, and other livestock throughout the year [3,4,5]. At the same time, under suitable water and fertilization conditions, it can form a dense turf layer with strong trampling resistance and regeneration ability, which constitutes the functional substrate of the alpine meadow ecosystem in the Tibetan Plateau [6]. However, this fragile ecosystem is facing multiple stresses, including the warming and drying trend caused by global climate change and frequent extreme weather events [7,8], soil erosion caused by the superposition of the *Ochotona curzoniae* cave system [9], and human overgrazing pressure [10], which has triggered a chain degradation effect. The data showed that the coverage of *C. alatauensis* in typical degraded areas dropped sharply from 85% to less than 30%, the biomass loss exceeded 70%, and the proportion of poisonous weeds increased to over 65% [1,11]. The succession of this plant community led to a decrease of 40–60% in organic matter content in the surface soil and a 50–70% decrease in available nitrogen, phosphorus, and potassium. This eventually formed the degradation pattern of “black soil beach”, where the vegetation–soil system was interrupted, rendering the ecosystem service functions irreversible [11].

Because of its extreme geographical environment and climate characteristics, alpine meadow ecosystems present obvious self-repair limitations. Studies have shown that the degraded “black soil beach” can hardly be restored to native meadow conditions within 100 years under natural succession conditions, and there is an urgent need to accelerate the process of ecological reconstruction relying on artificial intervention techniques [12,13]. Long-term efficacy assessments of artificially established grasslands reveal that during the initial vegetation restoration phase (1–3 years), vegetation coverage exceeding 85% can be achieved through appropriate plant configuration and soil improvement measures; however, upon entering the mid-term phase (3–5 years), community stability significantly declines, with a trend toward species monotonization. When the utilization period extends to 5–8 years, under the combined pressures of overgrazing and inadequate management, plant communities undergo retrogressive succession, resulting in an exponential decline in ecosystem service functions [14,15]. This phased decline characteristic of artificial grasslands reflects the dynamic imbalance between the biological regulatory mechanisms and the intensity of anthropogenic interventions in the process of alpine grassland restoration.

In light of this, with the large-scale application of perennial Poaceae forage restoration technology in “black soil beach” degradation remediation projects, the sustainable utilization of artificial grassland ecosystems has emerged as a critical bottleneck restricting the effectiveness of alpine meadow conservation and restoration efforts. At present, the core scientific problem to be solved urgently is as follows: can the artificial vegetation reconstruction model dominated by Poaceae effectively drive the positive succession process of degraded grasslands to their original alpine meadow state? To address this, this study focused on the population dynamics of *C. alatauensis*, a key constructive species of alpine meadow, taking the degraded grassland in the typical “black soil beach” of the Qinghai–Tibet Plateau as the objects. Through systematic analysis of its spatial distribution patterns, population expansion mechanisms, biomass accumulation dynamics, sexual/asexual reproductive strategies, and community succession associations, this research aimed to reveal the community succession pathways and driving mechanisms of artificial grasslands under moderate utilization intensity from the perspective of plant population ecology. The research provides theoretical support for constructing a technical paradigm of degraded grassland management based on a combination of natural restoration and artificial regulation and ultimately serves a scientific decision-making and effectiveness improvement for alpine grassland ecological restoration projects in the Qinghai–Tibet Plateau.

## 2. Results

### 2.1. Tillering Structure Parameters of C. alatauensis Under Different Restoration Years

#### 2.1.1. Characteristics of Tillers, Reproductive Shoots and Vegetative Shoots of *C. alatauensis* Under Different Restoration Years

**Figure 1:**
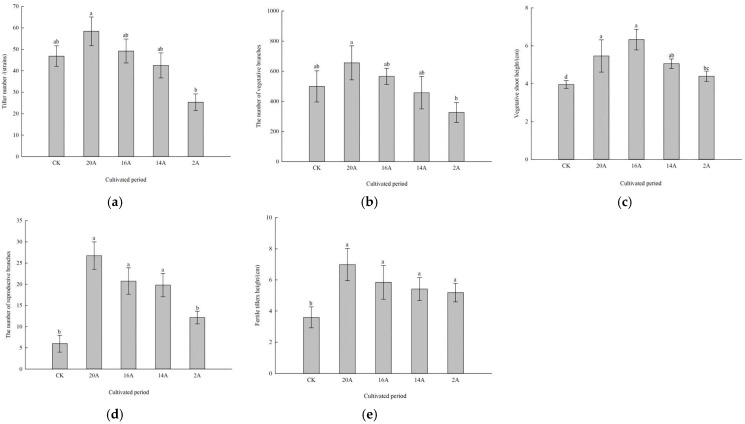
Changes in tillers, reproductive shoots and vegetative shoots of *C. alatauensis*. Note: Different lowercase letters indicate significant differences at the level of *p* < 0.05. (**a**) Tiller number; (**b**) the number of vegetative branches; (**c**) vegetative shoot height; (**d**) the number of reproductive branches; (**e**) fertile tiller height.

The number of tillers in *C. alatauensis* patches with different planting years is shown in Figure 1a. The number of tillers in the treatment of 20A reached the peak, and the number of tillers increased by 20.28%, 46.96%, 163.38% and 31.69%, respectively, compared with the treatment of 16A, 14A, 2A and CK. In terms of the number of vegetative shoots (Figure 1b), the treatments of 20A and 16A increased by 31.19% and 13.40%, respectively, compared with CK, and the treatment of 20A peaked. Compared with other treatment ages, the 20A treatment demonstrated increases of 15.69%, 43.33%, and 100.97% in vegetative shoot numbers relative to the 16A, 14A, and 2A treatments, respectively. A significant difference was observed between the 20A and 2A treatments (*p* < 0.05). Regarding vegetative shoot height (Figure 1c), all establishment age treatments significantly exceeded the CK (*p* < 0.05), with height increments ranging from 11.13% to 60.01%. Notably, the 16A treatment had the highest vegetative shoot height, which was increased by 43.99% compared to the 2A treatment, with a significant difference (*p* < 0.05).

In terms of the number of reproductive shoots (Figure 1d), the treatments with different planting years increased exponentially compared with the CK, among which the treatments of 20A, 16A and 14A significantly increased by 345.83%, 245.83% and 230.00%, respectively (*p* < 0.05), and the increase in 2A treatment also reached 102.78%. The comparison between years showed that the number of reproductive shoots in treatment 20A peaked, which increased by 28.92%, 35.10% and 119.86%, respectively, compared with treatment 16A, 14A and 2A, thus forming a succession pattern over time that increased with the extension of planting years. The reproductive shoot height demonstrated comparable succession trends (Figure 1e). All experimental treatments exhibited significant increases compared to CK (*p* < 0.05), with the magnitude of increase showing a gradient-based progression correlated with prolonged establishment age: the 20A treatment achieved a 94.43% increment, followed by 16A (62.54%), 14A (50.81%), and 2A (4.32%). A comparison between years showed that the height of the reproductive shoots of 20A treatment was increased by 19.62, 28.92 and 34.72% compared to 16A, 14A and 2A treatments, respectively, and there was no significant difference between treatments (*p* > 0.05).

#### 2.1.2. Spatial Distribution and Expansion Characteristics of *C. alatauensis* Patches in Artificial Grasslands with Different Establishment Ages

**Figure 2:**
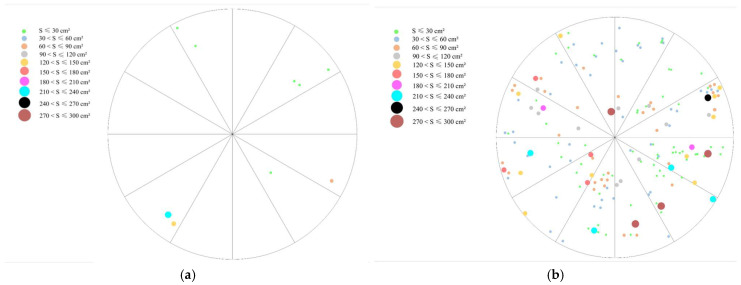

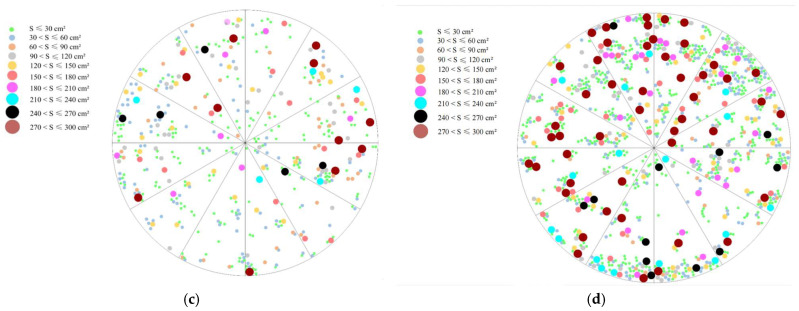
Individual distribution map and tussock structure of *C. alatauensis*. Note: (**a**) 2A; (**b**) 14A; (**c**) 16A; (**d**) 20A.

Analysis of the spatial patterns of *C. alatauensis* patches within sample circles (Figure 2) demonstrated significant heterogeneity in patch area. This study revealed significant temporal dynamics in the development of *C. alatauensis* patches during the succession of artificial grasslands: as restoration years increased, both the number and area distribution of the patches underwent a regular evolutionary trend. Their spatial distribution exhibited a pronounced aggregated pattern. Typical distribution patterns showed that large patches served as core areas, surrounded by small patches of varying sizes. As the restoration process advanced, the degree of patch aggregation continued to intensify, forming a nested spatial structure centered on dominant patches.

Based on monitoring data from 2019 to 2020 from artificial grasslands at different successional stages (Table 1), the patch structure of *C. alatauensis* exhibited significant interannual dynamics. Under CK, the proportion of small patches (≤90 cm^2^) increased markedly by 4.22%, while medium patches (90–180 cm^2^) expanded by 3.05%, and large patches (>180 cm^2^) decreased by 4.97%, indicating a patch fragmentation trend during natural succession. Conversely, 20A treatment demonstrated the opposite trajectory: small patches increased by 3.06%, large patches increased by 2.25%, and medium patches decreased by 2.73%, indicating that the system was developing towards a polarized patch structure. The 16A treatment showed a 2.09% increase in small patches and a marginal 1.54% rise in large patches, accompanied by a significant 3.93% reduction in medium patches, suggesting that the system was developing towards a polarized patch structure. In 14A treatment, small patches increased by 2.04% and large patches increased by 3.40%, while medium patches decreased by 4.04%, further reflecting a polarized structural shift.

#### 2.1.3. Biomass Characteristics of *C. alatauensis* Across Different Restoration Ages

**Figure 3:**
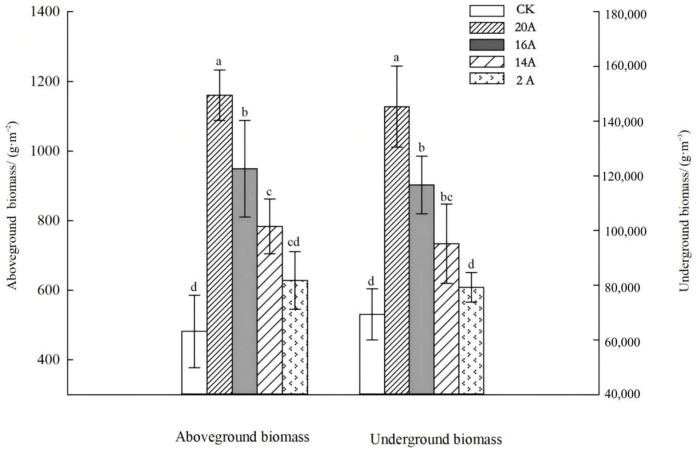
Changes in aboveground and underground biomass per unit area of *C. alatauensis*. Note: Different lowercase letters between the same index indicate that there are significant differences at the level of *p* < 0.05.

Systematic monitoring at different restoration ages revealed significant age-dependent patterns in the biomass accumulation of *C. alatauensis* patches (Figure 3). Aboveground biomass per unit area of artificial grassland patches increased in a gradient manner with restoration years, following the successional sequence 20A > 16A > 14A > 2A > CK. Statistical analysis indicated that aboveground biomass under 20A, 16A, and 14A treatments was significantly higher than CK, with increases of 140.90%, 97.02%, and 62.58% (*p* < 0.05), respectively, whereas 2A did not differ from CK (*p* > 0.05).

Belowground biomass dynamics were strongly coupled with aboveground components. This biomass accumulation pattern confirms that, during succession, *C. alatauensis* populations enhance resource acquisition capacity at the individual to population levels through synergistic interactions between root development and shoot growth. The 20A treatment exhibited the highest belowground biomass (145,264.71 g·m^−3^), followed by 16A (116,651.74 g·m^−3^), with both treatments significantly exceeding CK by 83.46% and 43.33%, respectively (*p* < 0.05).

### 2.2. Community Structure and Diversity Characteristics Under Different Restoration Years

#### 2.2.1. Plant Community Height, Coverage and Aboveground Biomass Characteristics

**Figure 4:**
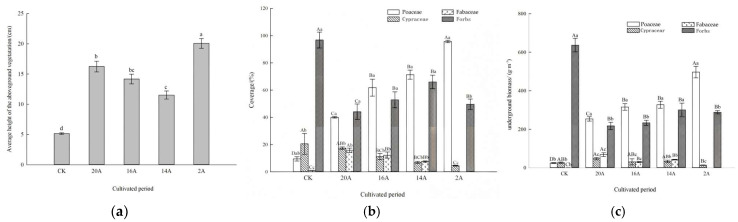
Changes in plant community height, coverage and biomass. Note: Different lowercase letters indicate significant differences at the level of *p* < 0.05 (**a**). Different uppercase letters in the same legend indicate significant differences at the level of *p* < 0.05, and different lowercase letters in the same treatment indicate significant differences at the level of *p* < 0.05 (**b**,**c**). (**a**) Average height of the aboveground vegetation; (**b**) coverage; (**c**) underground biomass.

During the vegetation restoration process of artificial grasslands, the height, coverage, and aboveground biomass of plant communities exhibited dynamic evolutionary patterns with prolonged restoration ages (Figure 4). Community Height Dynamics (Figure 4a) showed that all restoration treatments (2A, 14A, 16A, 20A) had significantly greater heights (20.08 cm, 11.53 cm, 14.19 cm, 16.25 cm) compared to CK (*p* < 0.05), with increases of 289.34%, 123.54%, 175.00%, and 215.06%, respectively. It is noteworthy that 2A grassland showed a distinct growth burst effect, and its height index was significantly higher than other treatments (*p* < 0.05).

In terms of coverage dynamics (Figure 4b), Poaceae dominated all restoration stages. Coverage levels for 20A (40.00%), 16A (61.83%), 14A (71.33%), and 2A (95.67%) significantly exceeded CK (10.00%) (*p* < 0.05), with increases of 300.00%, 518.30%, 613.33%, and 856.70%, respectively. Fabaceae coverage was 15.13% (20A), 12.25% (16A), 8.00% (14A), and 0.00% (2A), with all except 2A exceeding CK significantly (*p* < 0.05). Cyperaceae coverage in 16A (11.17%), 14A (6.87%), and 2A (4.50%) was significantly lower than CK (*p* < 0.05). Forb coverage was 44.06% (20A), 52.83% (16A), 66.03% (14A), and 44.61% (2A) (*p* < 0.05).

Aboveground Biomass Composition of Plant Communities (Figure 4c): Compared with CK, the biomass of Poaceae plants in 20A (253.81 g·m^−3^), 16A (315.61 g·m^−3^), 14A (327.28 g·m^−3^) and 2A (496.88 g·m^−3^) increased by 918.09%, 1165.98%, 1212.80% and 1893.10%, respectively (*p* < 0.05). Fabaceae biomass peaked at the 20A treatment, significantly exceeding other treatments and CK (*p* < 0.05). Cyperaceae aboveground biomass measured 47.19 g·m^−3^, 30.87 g·m^−3^, 33.13 g·m^−3^, and 0.00 g·m^−3^ in 20A, 16A, 14A, and 2A treatments, respectively, with all except 2A showing significant increases compared to CK (*p* < 0.05). The biomass of forb was 217.67 g·m^−3^, 233.20 g·m^−3^, 300.13 g·m^−3^ and 288.11 g·m^−^^3^ in treatments 20A, 16A, 14A and 2A, respectively, significantly lower than CK (*p* < 0.05).

#### 2.2.2. Characteristics of Grassland Plant Diversity

Community diversity succession (Table 2), artificial grassland and CK showed different diversity dynamics at different restoration stages. The CK exhibited the highest species richness (21.33), Simpson’s index (0.93), Shannon–Wiener index (2.78), and Pielou’s evenness index (0.91). Artificial grassland data showed that with increasing restoration age, the four diversity indexes all showed a gradual increasing trend. The 20A treatment demonstrated species richness of 20.67, which was 6.93%, 8.79%, and 10.71% higher than the 16A (19.33), 14A (19.00), and 2A (18.67) treatments, respectively. For diversity indexes, the Simpson index (0.90) of the 20A treatment increased by 7.14–11.11% compared with other recovery stages, and the Shannon–Wiener index (2.60) of the 20A treatment increased by 8.33–10.17% compared with other recovery stages. Pielou’s evenness (0.87) of the 20A treatment was 19.18% higher than 2A, and the difference was significant (*p* < 0.05).

Further analysis revealed significant differences between the four diversity indexes of artificial community at early establishment (treatment 2A) and CK (*p* < 0.05). Species richness, Simpson index, Shannon–Wiener index and Pielou evenness were significantly lower than CK (*p* < 0.05). With progress restoration, the four indexes of 20A grassland and short-term artificial community (2A, 14A and 16A) clearly increased, indicating long-term restoration effectively recovers the structural integrity of the community. This dynamic diversity of “initial decline-mid-adjustment-late restoration” confirms the self-repair potential and characteristics of the succession stages of an artificial grassland ecosystem.

### 2.3. Physicochemical Characteristics of Soil Under Different Restoration Years

#### 2.3.1. Soil pH Characteristics

**Figure 5:**
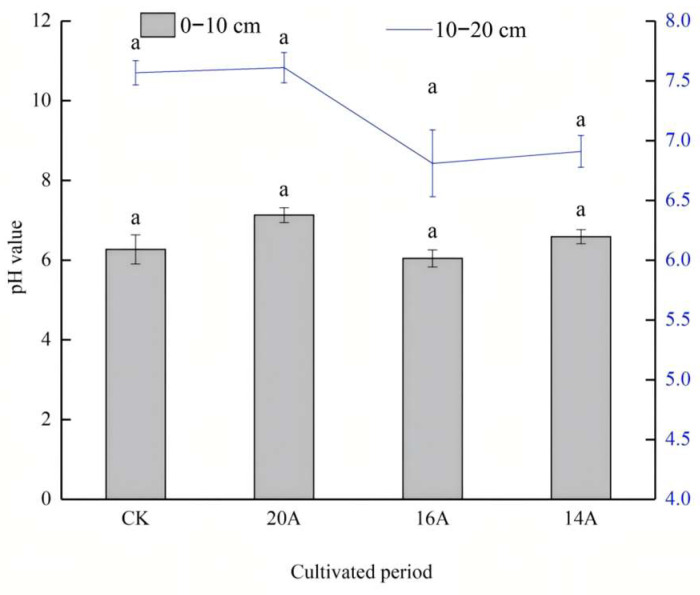
Changes in soil pH value. Note: Different lowercase letters in the same legend indicate significant differences at *p* < 0.05 level.

The soil pH varied with restoration years (Figure 5). In the 0–10 cm soil layer, soil pH increased by 13.7% in the 20A treatment and 5.1% in the 14A treatment compared to CK, neither of which reached significant levels (*p* > 0.05). The 20A treatment had 17.98% and 8.25% higher pH than 16A and 14A, respectively, but the differences were non-significant (*p* > 0.05). In the 10–20 cm soil layer, the 20A had the highest pH, with a 0.57% increase compared to CK, and 11.75% and 10.13% increases compared to 16A and 14A treatments, respectively. However, no significant differences occurred among treatments (*p* > 0.05).

#### 2.3.2. Characteristics of Soil Organic Carbon, Total Nitrogen and Total Phosphorus

**Figure 6:**
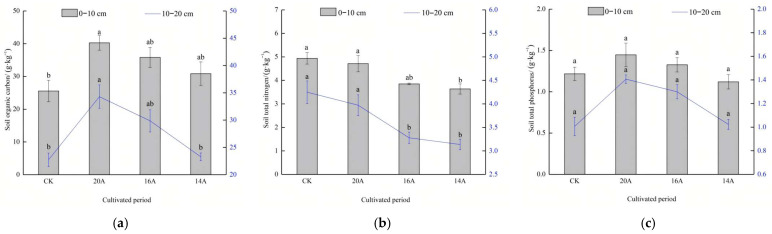
Changes in soil organic carbon, total nitrogen and total phosphorus. Note: Different lowercase letters in the same legend indicate significant differences in *p* < 0.05 level. (**a**) Soil organic carbon; (**b**) soil total nitrogen; (**c**) soil total phosphorus.

In the 0–10 cm soil layer, the SOC content exhibited a significant annual effect (Figure 6a). Specifically, the SOC content increased by 57.5%, 40.1% and 20.7% in the 20A, 16A and 14A treatments compared with CK, with only the difference between the 20A treatment and the CK reaching a significant level (*p* < 0.05). With increasing restoration age, the SOC content increased gradually. The 20A treatment was enhanced by 12.4% and 30.5% compared with the 16A and 14A treatments, respectively, though differences were non-significant (*p* > 0.05). In the 10–20 cm soil layer, SOC trends mirrored the surface layers, with the 20A, 16A and 14A treatments increasing by 50.9%, 31.4% and 2.3%, respectively, compared with the CK. The 20A treatment had the highest SOC content, which was 14.8% and 47.5% higher than the 16A and 14A treatments, respectively.

The distribution characteristics of the soil TN content are shown in Figure 6b. In the 0–10 cm soil layer, TN decreased by 4.6% (20A), 22.0% (16A), and 26.4% (14A), compared with that of CK, of which only the 14A treatment differed significantly from the CK (*p* < 0.05). Notably, compared with 16A and 14A treatments, the soil TN of 20A treatment increased by 22.4% and 29.7%, respectively. In the 10–20 cm soil layer, all treatments had lower TN than CK, but the 20A treatment increased by 21.1% and 26.7% compared with the 16A and 14A treatments, respectively, which was significant (*p* < 0.05).

The distribution characteristics of soil TP content are shown in Figure 6c. In the 0–10 cm soil layer, the soil TP content of the 20A and 16A treatments was elevated by 18.9% and 9.0%, respectively, compared with that of the CK, and the difference was not significant (*p* > 0.05). The 20A treatment increased by 9.1% and 29.2% compared to the 16A and 14A treatments, respectively, and the differences were not significant (*p* > 0.05). In the 10–20 cm soil layer, all treatments exceeded CK, and the TP content of treatment 20A was 8.2% and 37.5% higher than that of treatment 16A and 14A, respectively, with no significant difference (*p* > 0.05).

#### 2.3.3. Characteristics of Soil Ammonium Nitrogen, Nitrate Nitrogen and Available Phosphorus

**Figure 7:**
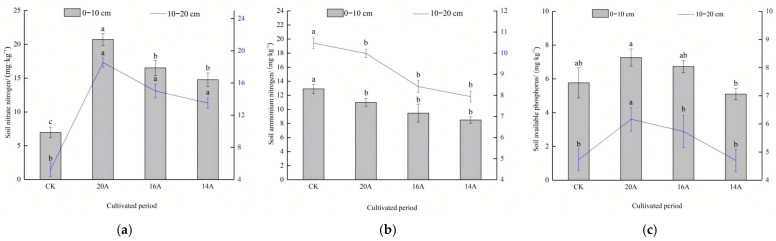
Changes in soil ammonium nitrogen, nitrate nitrogen and available phosphorus. Note: Different lowercase letters in the same legend indicate significant differences in *p* < 0.05 level. (**a**) Soil nitrate nitrogen; (**b**) soil ammonium nitrogen; (**c**) soil available phosphorus.

The distribution characteristics of the soil AN content are shown in Figure 7a. In the 0–10 cm soil layer, the AN content of the 20A, 16A and 14A treatments was reduced by 14.9%, 26.7% and 34.3%, respectively, compared with the CK (*p* < 0.05). Further analyses showed a steady increasing trend in AN content with increasing years of restoration, with the 20A treatment rebounding 16.0% and 29.5% (*p* > 0.05) compared to the 16A and 14A treatments, respectively. In the 10–20 cm soil layer, the contents of AN in treatments 20A, 16A and 14A were significantly lower than CK (*p* < 0.05). The 20A treatment increased by 18.6% and 25.7% (*p* > 0.05) compared to the 16A and 14A treatments, respectively, indicating that AN showed a slow accumulation trend in advanced restoration stages.

The characteristics of soil NN content changes are shown in Figure 7b. In the 0–10 cm soil layer, the NN content of the 20A, 16A, and 14A treatments was increased by 196.6%, 136.4%, and 111.4%, respectively, compared with that of the CK (*p* < 0.05), and the NN content of the 20A treatment was significantly increased by 125.4% and 40.3% compared with that of the 16A and 14A treatments (*p* < 0.05). In the 10–20 cm soil layer, the NN content of soil in 20A, 16A and 14A treatments significantly exceeded CK (*p* < 0.05); the NN content increased steadily with restoration age, with the NN content of the 20A treatment increasing by 23.6% and 37.3% compared with that of the 16A and 14A treatments, respectively, indicating that the NN content showed a continuous accumulation characteristic throughout restoration.

The characteristics of soil AP content changes are shown in Figure 7c. In the 0–10 cm soil layer, the AP content of 20A and 16A treatments increased by 26.0% and 16.8%, respectively, compared with that of CK (*p* > 0.05); the 20A treatment enhanced AP content by 7.9% and 42.5% compared to 16A and 14A. In the 10–20 cm soil layer, the AP content peaked at 20A, which was significantly higher (*p* < 0.05) than that in the other annual sample plots and CK, whereas there was no significant difference between the 16A and 14A treatments and the CK (*p* > 0.05).

### 2.4. Characteristics of C. alatauensis Tiller Structure Correlated with Plant Community Characteristics and Soil Physicochemical Properties

**Figure 8:**
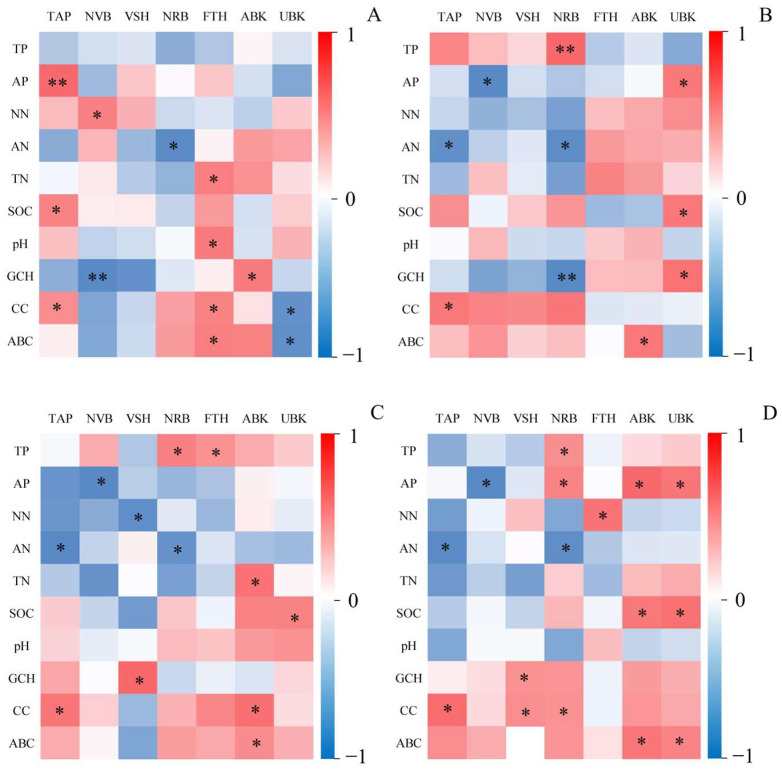
Correlation analysis between tillering structure of *C. alatauensis* and community structure, soil physicochemical properties. Note: (**A**) CK (Control treatment); (**B**) 20A; (**C**) 16A; (**D**) 14A. TAP: tillering number; NVB: number of vegetative branches; VSH: height of vegetative branches; NRB: number of reproductive shoot; FTH: height of reproductive shoots; ABK: aboveground biomass of *C. alatauensis*; UBK: belowground biomass of *C. alatauensis*; TN: total nitrogen; TP: total phosphorus; NN: nitrate nitrogen; AN: ammonium nitrogen; AP: available phosphorus; SOC: soil organic carbon; pH: soil pH; GCH: community height; CC: coverage of Cyperaceae; ABC: biomass of Cyperaceae. * *p* < 0.05; ** *p* < 0.01.

As shown in Figure 8A, there were 13 pairs of significant/significant correlations, 9 pairs of positive correlations and 4 pairs of negative correlations between the CK *C. alatauensis* tiller characteristics and plant community and soil traits. There was a significant positive correlation between tillering number (TAP) and SOC, coverage of Cyperaceae (CC) (*p* < 0.05), and a highly significant positive correlation between TAP and AP (*p* < 0.01). There was a significant positive correlation between the number of vegetative branches (NVB) and NN (*p* < 0.05) and a highly significant negative correlation between NVB and community height (GCH) (*p* < 0.01). There was a significant negative correlation between the number of reproductive shoots (NRB) and AN (*p* < 0.05). There was a significant positive correlation between the height of reproductive shoots (FTH) and TN, pH, CC, biomass of Cyperaceae (ABC) (*p* < 0.05). There was a significant positive correlation between the aboveground biomass of *C. alatauensis* (ABK) and GCH (*p* < 0.05). There was a significant negative correlation between the belowground biomass of *C. alatauensis* (UBK) and CC, ABC (*p* < 0.05).

As shown in Figure 8B, there were 10 pairs of significant and highly significant correlations, 5 pairs of positive correlations and 4 pairs of negative correlations between the tillering characteristics of *C. alatauensis* in the 20A treatment and plant community and soil traits. There was a significant positive correlation between TAP and CC (*p* < 0.05) and a significant negative correlation between TAP and AN (*p* < 0.05). There was a significant negative correlation between NVB and AP (*p* < 0.05). There was a significant negative correlation between NRB and AN (*p* < 0.05), a highly significant negative correlation between NRB and GCH (*p* < 0.01), and a highly significant positive correlation between NRB and TP (*p* < 0.01). There was a significant positive correlation between ABK and ABC (*p* < 0.05). There was a significant positive correlation between UBK and AP, SOC, GCH (*p* < 0.05).

As shown in Figure 8C, there were 12 pairs of significant/highly significant correlations, 8 pairs of positive correlations and 4 pairs of negative correlations between 16A-treated *C. alatauensis* tillering characteristics and plant community and soil traits. There was a significant positive correlation between TAP and CC (*p* < 0.05) and a significant negative correlation between TAP and AN (*p* < 0.05). There was a significant negative correlation between NVB and AP (*p* < 0.05). There was a significant positive correlation between the height of vegetative branches (VSH) and GCH (*p* < 0.05) and a statistically significant negative correlation between VSH and NN (*p* < 0.05). There was a statistically significant positive correlation between NRB and TP (*p* < 0.05) and a statistically significant negative correlation between NRB and AN (*p* < 0.05). There was a significant positive correlation between ABK and TN, CC, ABC (*p* < 0.05). There was a significant positive correlation between UBK and SOC (*p* < 0.05).

As shown in Figure 8D, there were 16 pairs of significant/highly significant correlations between the tillering characteristics of *C. alatauensis* and plant community and soil properties under the 14A treatment, 13 pairs of positive correlations and 3 pairs of negative correlations. A significant positive correlation was observed between TAP and CC (*p* < 0.05), while a significant negative correlation existed between TAP and AN (*p* < 0.05). The number of vegetative branches (NVB) exhibited a significant negative correlation with AP (*p* < 0.05). The height of vegetative branches (VSH) showed significant positive correlations with both GCH and CC (*p* < 0.05). The number of reproductive shoots (NRB) demonstrated a significant negative correlation with AN (*p* < 0.05), alongside significant positive correlations with TP, AP, and CC (*p* < 0.05). Both ABK and UBK displayed statistically significant positive correlations with AP, SOC, and ABC (*p* < 0.05).

## 3. Discussion

### 3.1. Reproductive and Expansion Characteristics of C. alatauensis

The population expansion of *C. alatauensis* is largely dependent on asexual reproduction mechanisms, with the number of tillers, vegetative shoots and reproductive shoots being the core indicators of reproductive success. This study showed that in degraded grassland, the number of tillers and vegetative shoots of *C. alatauensis* was significantly lower than that of artificial grassland established for 16 and 20 years, indicating that soil degradation and competition from poisonous weeds inhibited its reproduction, which is consistent with the findings of existing studies [3,16]. In the process of artificial grassland restoration, the implementation of optimal grass mixtures and supporting forbidden grazing management measures effectively suppressed the competitive intensity of poisonous weeds and optimized soil water and fertilization conditions, thus significantly accelerating the tillering process of *C. alatauensis* and enhancing the number of reproductive shoots, as well as strengthening the recovery potential of its sexual reproduction [17]. The number of tillers and the number and length of vegetative shoots and reproductive shoots showed a continuous increase with the number of years of restoration. At the same time, the dominance of grassy Poaceae plants such as pendulous lancelet had been decreasing year by year, creating more favorable conditions for *C. alatauensis* to access resources and further promoting the expansion of its tillers and meristems [18]. In addition, a ‘ring-like’ growth structure develops as the tussock ages, which is reflected by the increased vigor of the new branches at the outer edges and the gradual decline in the central branches. This phenomenon is particularly significant in perennial grasslands, revealing the survival strategy of *C. alatauensis* to achieve population renewal through the dynamic regulation of the clumping structure. Comprehensively speaking, the restoration measures of artificial grassland not only directly promote the reproduction of *C. alatauensis* by improving the environmental conditions but also significantly enhance its sexual reproduction ability and community self-renewal ability, which provides an important biological theoretical basis and practical pathways for the ecological restoration of degraded alpine meadows [17].

Based on the continuous observation data in 2019–2020, the patch size distribution of *C. alatauensis* in artificial grassland and degraded grassland differed significantly: Artificial grassland showed a gradient of ‘large patches > small patches > medium patches’, while the degraded grassland showed a reverse pattern of ‘large patches > medium patches > small patches’. Degraded grassland had a significantly lower proportion of small patches than artificial grassland and showed negative growth in total area since 2020, despite having a higher proportion of large patches. This patch dynamic echoes the findings of Xu et al. [19], revealing a continuous decline in the dominance of Cyperaceae communities during degradation and a successional pattern where poisonous weeds gradually occupy ecological niches. As a core grassland health indicator, the productivity level, community ecosystem function, and dynamic changes in biomass profoundly affect the spatial distribution pattern of species and community succession [20]. Compared to degraded grassland, artificial grassland increased the aboveground biomass by 30.40–140.90% and belowground biomass by 14.20–109.51%. This difference is mainly attributed to the severe wind and water erosion of degraded grasslands and the competitive effect of poisonous weeds on soil nutrients, which directly inhibit the growth and development of *C. alatauensis* populations by destroying the soil structure, resulting in the blockage of aboveground and belowground biomass accumulation [21,22]. It is worth noting that the artificial grassland of different restoration years showed the characteristics of significantly higher belowground biomass than aboveground biomass, which is consistent with the findings of Li et al. [22]. This phenomenon reveals the survival strategy of *C. alatauensis* populations under natural conditions: due to the limited ability of sexual reproduction, the populations safeguard the developmental needs of the aboveground parts by strengthening the material and energy reserves of the underground rhizome system and constructing a growth pattern based on nutrient enrichment. Further analyses showed that the number of years of restoration of the artificial grassland was significantly and positively correlated with the biomass accumulation of *C. alatauensis* grass patches. With an extension in planting time, the aboveground and belowground biomass per unit area exhibited a continuous increasing trend, which is consistent with the hypothesis of ‘populations stability drives biomass accumulation’ put forward by scholars such as Gao et al. [18] and Li et al. [22]. This confirms that artificial grassland can effectively promote the stable growth of *C. alatauensis* populations by extending the recovery cycle and then achieve the gradual recovery of ecosystem function.

### 3.2. Characteristics of Plant Community Structure and Diversity

This study systematically revealed differential pathways and ecological mechanisms of community succession during artificial grassland restoration by analyzing key indicators, such as plant community height, coverage, and biomass. In degraded grassland, the aggressive invasion of poisonous weeds has induced a “pseudo-stabilization” characteristic in plant communities. Although the absolute dominance of poisonous grasses enhances the stability of the community, the living space of the grasses of the Poaceae-Cyperaceae is significantly compressed, and the multifunctionality of the ecosystems has continued to decline, resulting in the evolution of a monocultural community structure dominated by poisonous weeds [23]. The ecosystems have been declining in multifunctionality. Conversely, the restoration of artificial grassland shows a clear law of stage succession: at the initial stage of establishment, perennial Poaceae plants such as *E. nutans* and *p. pratensis* establish rapidly, and the spread of forbs is effectively inhibited through the space occupation effect [24]; as restoration progresses, the niche advantage of Cyperaceae pasture gradually becomes prominent, and its population proportion continues to rise and eventually replace forbs, promoting the succession of community structure to Poaceae-Cyperaceae composite dominance, which significantly accelerates the ecological function reconstruction process of artificial grassland [25,26]. It is worth noting that *C. alatauensis*, as the primary dominant species in alpine meadow, its coverage and biomass ratio showed a continuous upward trend during restoration, which highlighted the core position of Cyperaceae plants in maintaining community functions.

The community diversity monitoring revealed that the species richness, Shannon–Wiener index, Simpson index and Pielou evenness index of the artificial grassland showed a stable increase with an extension in restoration years, reflecting that the species composition evolved in the direction of complexity and equilibrium [27]. This mechanism of diversity enhancement stems from two main aspects: firstly, the expansion of the ecological niche of plants in the Cyperaceae reinforces the vertical stratification effect of resource utilization [28]; secondly, the excellent forage grasses constructed a more stable interspecific relationship network through ecological niche differentiation, suppressing forb’s competitive advantage [29]. Although the diversity of degraded grassland increased temporarily due to the outbreak of forbs, the degradation of forage quality led to the serious degradation of ecosystem service capacity. Conversely, the artificial grassland successfully constructed a multifunctional and anti-interference composite community structure through the synergistic effect of excellent pasture combination and moderate grazing management, in agreement with the research conclusions of Wang et al. [30] and Luo et al. [31] in typical meadows on the Qinghai–Tibet Plateau. This study confirms that artificial grassland restoration formed a replicable alpine meadow degradation management model through Poaceae-Cyperaceae dominant species replacement, which provides an important theoretical reference for the functional reconstruction of similar degraded ecosystems around the world.

### 3.3. Physicochemical Characteristics of Artificial Grassland Soil and Its Synergistic Restoration Mechanism with Vegetation Cover

As the basic medium for vegetation growth and development, the physicochemical properties of soil play a decisive role in the maintenance of ecosystem function and the direction of community succession in alpine meadows [32]. In the degraded grassland of the ‘black soil beach’ type, the chain reaction of wind erosion and water erosion triggered by the sharp reduction in surface vegetation cover resulted in the key nutrient indexes of SOC, TP, NN and AP being significantly lower than that of the artificial grassland (*p* < 0.05), which was strongly consistent with the findings of studies on similar degraded meadows on the Qinghai–Tibetan Plateau [21,33,34,35]. The TN content in the 0–20 cm soil layers is abnormally high in the degraded area, which may be due to two mechanisms: first, the surface exposure caused by vegetation degradation intensifies the combined action of freeze–thaw and wind erosion, which promotes the migration of nitrogen in deep soil to the surface; secondly, in the process of the mineralization of plant residues at the end of the growing season, the decomposition inhibition effect of rhizomes with a high carbon–nitrogen ratio may change the nitrogen cycle path [36]. The restoration process of artificial grassland showed a significant soil–vegetation synergistic improvement: with an extension in the establishment period, the SOC, TN and TP contents accumulated continuously. The nutrient dynamics were closely related to the successional stage of the community. In the early stages of restoration, Poaceae plants develop a competitive advantage through rapid growth, and their high nitrogen demand characteristics lead to a relative lag in soil TN content [27,37]. As the restoration process advances, the proportion of plants of Cyperaceae (*C. alatauensis*) and Fabaceae increases, and this dominant family turnover reshapes soil nutrient cycling in three ways: (1) lowering the average carbon to nitrogen ratio of the community and promoting the mineralization of plant residues; (2) enhancing the role of inter-root sedimentation and expanding the channels for organic matter input; (3) optimizing the microbial community structure and accelerating nutrient turnover efficiency [34,38].

In this study, the accumulation rates of soil TP and AN, NN were significantly higher than expected from previous studies [30], and this difference may be related to the grazing management system specific to the region. The pattern of ‘no grazing in the growing season + winter grazing’ adopted in the experimental plots regulated the aboveground and belowground biomass distribution pattern through moderate disturbance; livestock grazing changed the spatial distribution characteristics of the dominant species and prompted the plant root system to extend to deeper soil, and this compensatory growth strategy enhanced the activation and absorption capacity of phosphorus [39]. At the same time, the input of organic debris from winter grazing creates a slow-release nutrient pool at low temperatures, and this ‘seasonal pulse’ pattern of nutrient supply may be a key driver of soil nitrogen accumulation [39]. Correlation analyses of *C. alatauensis’s* tiller structure with plant community and soil physicochemical properties showed a significant positive correlation between both understory biomass (UBK) and SOC in *C. alatauensis* grass. This is similar to previous findings in alpine and sub-alpine grasslands [13,40,41], in that soil nutrient addition improves plant growth and reproduction and, hence, community structure. Although the soil quality of artificial grassland showed a progressive improvement with years of restoration, the rate of community succession was still limited by the stage of soil development. Data analysis showed that the soil TN content of artificial grassland 20 years after planting was still 18.7% lower than natural grassland, suggesting that a longer time scale is required for full natural recovery. Therefore, it is suggested to adopt the management strategy of “regulation by stages”: in the early stage of restoration (0–5 years), the whole period of grazing prohibition should be implemented to promote vegetation coverage; in the middle stage (5–15 years), moderate rotational grazing should be introduced to maintain community diversity; and in the later stage (15 years later), the reconstruction of the SOC pool should be accelerated with the application of organic fertilizer [21,35,38]. This dynamic management scheme can provide a more operational practical path for the ecological restoration of degraded alpine meadows by coupling biological processes and soil development laws.

## 4. Materials and Methods

### 4.1. General Situation of Test Site

**Figure 9:**
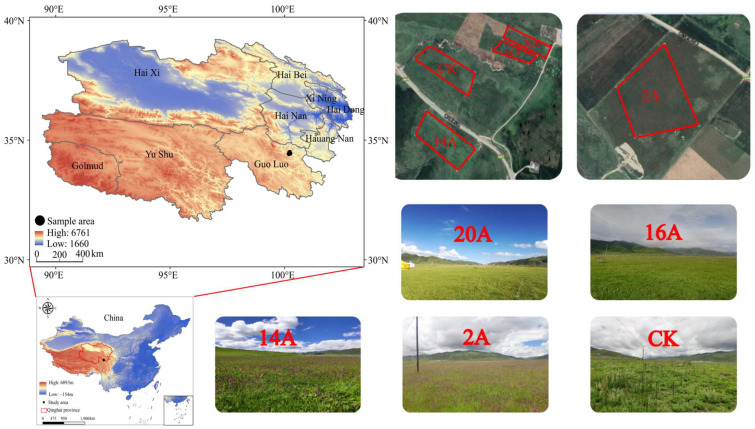
Geographical location of test plot and sampling site.

The study area is situated in Maqin County, Qinghai Province, on the eastern margin of the Qinghai–Tibet Plateau, China (Figure 9), with an average elevation of 3760 m. The region belongs to a typical plateau continental climate, with annual precipitation ranging from 513.2 mm to 542.9 mm and an average annual temperature of −3.9 °C. Climatic constraints result in relatively short vegetation growth periods, typically lasting 110 to 150 days. The soil in the study area is predominantly classified as Alpine Meadow Soil, with significant surface organic matter accumulation, exhibiting typical characteristics of an alpine meadow ecosystem [40].

The vegetation community exhibits vertical differentiation with dominant species comprising the graminoid layer formed by *Elymus nutans* Griseb. (Poaceae) and *Poa araratica* Trautv. (Poaceae); the dense cushion-forming layer composed of *C. alatauensis *(Cyperaceae) and *Carex parvula* O. Yano (Cyperaceae); the forb layer represented by *Pedicularis kansuensis* Maxim. (Orobanchaceae), *Aconitum pendulum* Busch (Ranunculaceae), and *Ligularia virgaurea* Mattf. (Asteraceae). This structural organization reflects the adaptive characteristics of alpine meadow ecosystems to low temperatures and short growing seasons, forming a unique vertical ecological sequence [41].

### 4.2. Experimental Design

In this study, the field investigation was carried out in the peak season of plant growth in 2019 and 2020, and the representative research plots were selected according to the principles of topographic consistency, geomorphological consistency and spatial proximity. The experimental design comprised four artificial grassland treatments with varying establishment ages (20-year artificial grassland established in 2000, 16-year artificial grassland established in 2004, 14-year artificial grassland established in 2006 and 2-year artificial grassland established in 2018), alongside the degraded grassland as the control treatment (CK). Winter grazing management (November to April each year) was implemented in all experimental plots, and a complete grazing ban was imposed during the growing season. The basic information for the sample plots is detailed in Table 3, and the spatial configuration and layout of sampling sites are shown in Figure 9.

Based on the principle of random distribution, three circular observation quadrats with a diameter of 30 m (with a single quadrat area of 707 m^2^) were set up in each treatment plot, and the functional characteristics of *C. alatauensis* were systematically determined. The specific observation indicators include the following: (1) Patch characteristics of *C. alatauensis* populations: to measure the patch area of *C. alatauensis* using its geometric characteristics, calculate the coverage of *C. alatauensis* patches, and record the number of patches. (2) Biomass index: the existing aboveground biomass and underground root biomass of *C. alatauensis* community were measured at different depths. (3) Morphological characteristics of plants: the number of tillers, vegetative shoots and reproductive shoots per unit area was counted, and the vertical growth height of each type of branch was measured simultaneously. Within each treatment sample plot, six 1 × 1 m sample squares were set up using a randomized deployment scheme, with a total of 30 sample squares, to systematically carry out the determination of the characteristics of the *C. alatauensis* community. Specific observation indicators included plant species, vegetation cover and plant height and aboveground biomass.

### 4.3. Index Determination and Methods

#### 4.3.1. Functional Characteristics of *C. alatauensis*

**Figure 10:**
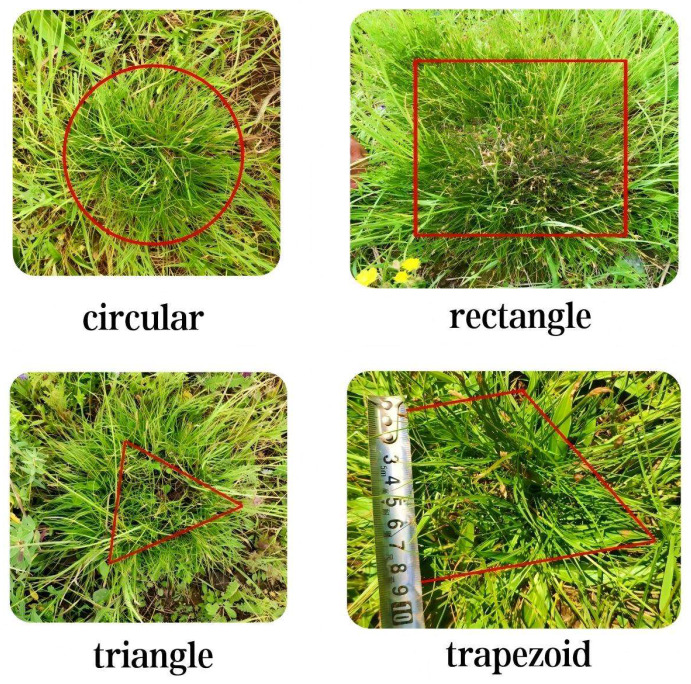
Determination of patch area of *C. alatauensis*.

Determination of patch area. (1) Shape classification: based on the geometric characteristics of target patches within circular sample plots, patches were categorized into four types (circular, triangular, rectangular, and trapezoidal (Figure 10)). (2) Determination of parameters: for the approximate triangular patches of *C. alatauensis*, the length of the bottom edge and the corresponding height were measured using a steel ruler with an accuracy of 1 mm. The area was calculated using the formula area = bottom edge length × height/2; for the patches approximating rectangular shapes, the lengths of the longest and shortest perpendicular sides within the patch were measured. The area was calculated using the formula: area = length × width. (3) Irregular patch processing: for patches with complex shapes, they were first broken down into several regular geometric shapes (circles, triangles, rectangles), then the areas were measured separately, and finally summed.

Patches were subdivided and classified into 10 categories based on a 30 cm^2^ area gradient: ① extremely small patches (S ≤ 30 cm^2^), ② 30–60 cm^2^, ③ 60–90 cm^2^, ④ 90–120 cm^2^, ⑤ 120–150 cm^2^, ⑥ 150–180 cm^2^, ⑦ 180–210 cm^2^, ⑧ 210–240 cm^2^, ⑨ 240–270 cm^2^, and ⑩ extra-large patches (S > 270 cm^2^).

Determination of biomass and distribution of *C. alatauensis*. (1) Patch Distribution Survey: the spatial coordinates and coverage of *C. alatauensis* patches in the sample circle were recorded via grid positioning method, and the spatial distribution pattern of *C. alatauensis* population was obtained. (2) Aboveground biomass determination: Aboveground vegetation of target patches was clipped at ground level using sharp pruning shears. Immediate weighing was performed with an electronic balance (0.01 g precision). Fresh samples were placed in breathable storage bags, transported to the laboratory on the same day, and subjected to oven drying at 105 °C for 30 min; forced-air drying at 80 °C for 24 h, with dry mass considered stable when consecutive weighing showed ≤0.005 g difference; final dry biomass was quantified using an analytical balance (0.0001 g precision). (3) Belowground biomass determination: At the corresponding coordinate point where the aboveground part was sampled, the root system was collected with a root drill (φ = 7 cm), and the root system was separated from soil particles with a grading sieve. After being rinsed with deionized water, the dry sample of the underground part was prepared using the same drying procedure as that of the aboveground part [42].

Determination of tillering characteristics. Complete plants were examined under a dissecting microscope to identify ramets and differentiate reproductive shoots from vegetative shoots. Reproductive shoots were defined as individuals bearing mature inflorescences or exhibiting floral bud differentiation. The number of reproductive and vegetative shoots was recorded, and their vertical heights were measured using digital calipers (0.01 mm precision).

#### 4.3.2. Community Characteristics of *C. alatauensis*

Investigating plant community species composition, plant height, number of species, species coverage, total community cover, aboveground biomass and belowground biomass. The biomass measurement method is the same as in Section 4.3.1 [42]. The richness of categories is expressed by the number of species in the quadrat; the average plant height of a species represents the plant height of that species; species coverage and total community coverage were measured using visual method and expressed as the average of six quadrats. Simpson diversity index (D), Shannon–Wiener diversity index (H), Pielou evenness index (E) and Species richness index (R) are selected as four plant diversity indexes, and the calculation formula is as follows:

(1) Simpson’s diversity index (D): D = 1 − ΣP***_i_***^2^
(2) Shannon–Wiener diversity index (H): H = −ΣP***_i_***lnp***_i_***
(3) Pielou’s evenness index (E): E = −ΣP***_i_***lnp***_i_***/lnS
(4) Species richness index (R): R = total number of observed species in the plot

P***_i_*** = proportion of individuals belonging to species ***_i_*** relative to total individuals in the plot; S = total number of species in the community.

#### 4.3.3. Soil Characteristics

Six drills of 0~10 cm and 10~20 cm soil layers were randomly drilled in the quadrat with a soil drill with a diameter of 3.8 cm. The soil samples were mixed well, and then gravel and plant residues were picked out and passed through a 2 mm sieve for determination of soil nutrient content (soil organic carbon SOC, total nitrogen TN, total phosphorus TP, nitrate nitrogen NN, ammonium nitrogen AN and available phosphorus AP) and pH value [43,44]. The determination method refers to Soil Agrochemical Analysis (Third Edition) [43,44].

### 4.4. Data Analysis Methods

Data were organized using Microsoft Excel 2016. Statistical analyses of plant community height, coverage, biomass, and soil physicochemical properties in artificial grasslands of different establishment ages in black soil beach areas were performed using IBM SPSS Statistics 22.0 with one-way analysis of variance (ANOVA). Data were presented as mean ± standard deviation (SD), with statistical significance set at *p* < 0.05. Graphs including bar charts, spatial pattern diagrams, and correlation heatmaps were generated using Origin 2017 and SigmaPlot 14.0.

## 5. Conclusions

This study systematically revealed the succession mechanism of an alpine meadow driven by *C. alatauensis* population expansion through long-term positioning observations of artificial grasslands with different establishment ages on the Qinghai–Tibet Plateau. The main conclusions are as follows: (1) Prolonged establishment of artificial grasslands significantly promoted the asexual reproduction of *C. alatauensis* (tiller number, vegetative shoots, and reproductive shoots showed sustained growth). Underground biomass increased with establishment age, with the underground biomass per unit area in 20A artificial grassland being significantly higher than that in 2A grassland (2A) (*p* < 0.05). (2) *C. alatauensis* patches exhibited aggregated distribution patterns, with large patches dominating. As restoration progressed, small new patches around large patches significantly increased, forming a spatial expansion trend. (3) The vertical structure of plant communities was optimized during restoration. The coverage and biomass proportions of Cyperaceae and Fabaceae plants increased annually, while the dominance of Poaceae and forbs declined. Species richness, Shannon–Wiener index, Simpson index, and Pielou evenness index significantly increased with restoration duration (*p* < 0.05), indicating a succession trend toward the natural meadow structure. (4) Nutrient contents in the 0–20 cm soil layer showed positive correlations with establishment age. Soil organic carbon, total phosphorus, and available phosphorus contents in 20A artificial grassland were significantly higher than those in 2A grassland and degraded grassland (CK) (*p* < 0.05). Correlation analysis revealed significant/highly significant relationships between the tillering structure of *C. alatauensis* and plant community characteristics/soil physicochemical properties. In summary, artificial grassland restoration optimizes soil environments and regulates species composition, forming a stable community structure dominated by *C. alatauensis*. This study provides a scientific paradigm of “soil-vegetation” synergistic restoration for degraded alpine meadow management.

## Figures and Tables

**Table 1:** Patch proportion of small, medium and large *C. alatauensis*.

Sample Plot Number	Patch Type	Proportion%		Growth Rate%
		2019	2020	
CK	Small	14.23	14.83	4.22
	Middle	18.58	19.15	3.05
	Large	67.19	63.85	−4.97
20A	Small	31.15	32.10	3.06
	Middle	20.08	19.53	−2.73
	Large	48.77	49.87	2.25
16A	Small	32.31	32.09	2.09
	Middle	20.46	19.65	−3.93
	Large	47.23	47.96	1.54
14A	Small	33.23	33.91	2.04
	Middle	22.84	21.91	−4.04
	Large	43.94	45.43	3.40
2A	Small	—	—	—
	Middle	—	—	—
	Large	—	—	—

Note: Small patches S ≤ 90 cm^2^, middle patches 90 cm^2^ < S ≤ 180 cm^2^ and large patches S > 180 cm^2^.

**Table 2:** Changes in plant community diversity in artificial grassland with different planting years.

Serial Number of Plots	Species Richness	Simpson Index	Shannon–Wiener Index	Pielou Index
CK	21.33 ± 1.11 a	0.93 ± 0.01 a	2.78 ± 0.02 a	0.91 ± 0.02 a
20A	20.67 ± 2.89 a	0.90 ± 0.01 a	2.60 ± 0.17 a	0.87 ± 0.01 a
16A	19.33 ± 1.11 a	0.84 ± 0.04 a	2.40 ± 0.10 a	0.81 ± 0.04 a
14A	19.00 ± 3.33 a	0.83 ± 0.07 a	2.39 ± 0.25 a	0.79 ± 0.06 a
2A	18.67 ± 0.44 b	0.80 ± 0.02 b	2.36 ± 0.19 b	0.73 ± 0.07 b

Note: Different lowercase letters in the same column indicate that the level of 0.05 is significantly different.

**Table 3:** Basic information of study plot.

Establishment Year	Establishment Duration	Altitude /m	Longitude and Latitude	Dominant Species
degraded grassland (CK)	0A	3727	100°12′41.2″ E 34°27′53.2″ N	*L. Virgaurea*, *Artemisia hedinii* Ostenf., *A. pendulum*
2000	20A	3735	100°13′5.6″ E 34°27′59.6″ N	*E. Nutans, C. alatauensis*, *C. parvula*
2004	16A	3735	100°13′5.3″ E 34°27′59.0″ N	*E. nutans, P. crymophila*, *C. parvula*
2006	14A	3747	100°12′34.4″ E 34°27′38.8″ N	*E. Nutans*, *Festuca ovina* L., *A. pendulum*
2018	2A	3720	100°15′1.73″ E 34°26′9.86″ N	*E. Nutans*, *P. kansuensis*, *Lancea tibetica* Hook. f. & Thomson

## Data Availability

The original contributions presented in the study are included in the article; further inquiries can be directed to the corresponding authors.
